# Using routinely collected patient data to study the impact of type 2 diabetes on breast cancer

**DOI:** 10.1530/EO-24-0039

**Published:** 2025-07-08

**Authors:** Ayaan Khurshed, Gema Hernandez, Gavin Soady, Nalinie Joharatnam-Hogan, Daniel Morganstein

**Affiliations:** ^1^ Beta Cell Diabetes Unit, Chelsea and Westminster NHS Foundation Trust, London, UK; ^2^ TriNetX, Cambridge, Massachusetts, USA; ^3^ Breast Unit, Royal Marsden Hospital NHS Foundation Trust, London, UK

**Keywords:** breast cancer, diabetes, survival, adjuvant chemotherapy

## Abstract

**Objective:**

Type 2 diabetes mellitus (T2DM) and cancer are prevalent conditions, with evidence linking T2DM to higher breast cancer incidence and mortality. However, it is uncertain whether excess mortality in breast cancer patients with diabetes is driven by cancer-related factors. This study aims to investigate overall survival (OS) and chemotherapy receipt post-surgery in women with diabetes and localised breast cancer.

**Methods:**

Cohorts were constructed from electronic patient records on TriNetX, a de-identified patient database. Three cohorts included women with diabetes stratified by localised breast cancer stage (1 & 2, 3 and all), compared to control groups without diabetes. Cohorts were propensity score matched for age, ethnicity, smoking status and BMI. OS and chemotherapy receipt were compared.

**Results:**

Patients with diabetes (*n* = 1,488) were significantly older, more likely to smoke and had higher BMIs than those without diabetes (*n* = 7,284). The unadjusted hazard ratio (HR) for OS across all cancer stages was 2.17 (95% CI: 1.90–2.485) and the adjusted HR was 1.69 (95% CI: 1.41–2.04). After further adjusting for vascular diseases, the HR for OS was 1.59 (95% CI: 1.32–1.92). No significant difference was found in chemotherapy receipt.

**Conclusion:**

We observed significantly poorer OS in women with breast cancer and diabetes across all stages, compared to those without diabetes. Importantly, this persisted after adjusting for confounders and cardiovascular diseases, supporting that diabetes directly influences cancer outcomes.

## Background

Type 2 diabetes and breast cancer are both common conditions, and there is increasing evidence that breast cancer occurs more frequently in those with type 2 diabetes (Ling *et al.* 2020).

Diabetes seems to have a role not only in the development of cancer but also has adverse effects on cancer outcomes. A systematic review and meta-analysis of 23 studies looking at various types of cancer shows that cancer patients with pre-existing diabetes at the time of diagnosis have a 41% higher risk of mortality than those without diabetes (Barone *et al.* 2008). Hyperglycaemia itself has been linked to poorer overall and disease-free survival in cancer patients, as supported by one meta-analysis of 12 studies, which showed double the hazard ratios (HRs) for both measurements (Barua *et al.* 2018).

Focusing on breast cancer, patients with T2DM experience poorer outcomes compared to controls without diabetes (summarised in Supplementary Table 1 (see section on Supplementary materials given at the end of the article)). Some research shows that disease-free survival and overall survival (OS) are significantly shorter in patients with diabetes and breast cancer (Mu *et al.* 2017, Sonnenblick *et al.* 2017, Lee *et al.* 2019). Others report all-cause mortality HRs ranging from 1.11 to 3.65 for breast cancer patients with T2DM (Chang *et al.* 2018, Lega *et al.* 2018, Tao *et al.* 2020). Cancer-specific mortality showed a HR of 1.10 (Escala-Garcia *et al.* 2020), while another study highlighted a lower 5-year survival rate for patients with diabetes (Lawrenson *et al.* 2024). However, data on cancer outcomes in patients with T2DM vary substantially across the literature, likely due to inconsistencies in including the cause of death, differences in accounting for cancer stage, varying definitions of diabetes, or matching of controls across studies, and heterogeneity in population demographics.

Women with diabetes tend to present with later-stage breast cancers, even if they are regularly screened (Lipscombe *et al.* 2015). Patients with T2DM may also face a higher incidence of cancer treatment modifications than patients without diabetes. Another disparity in cancer management was found in a study that analysed the treatment and outcomes of 58,498 cancer patients, which reported that less aggressive treatment was given to patients with T2DM (van de Poll-Franse *et al.* 2007). For instance, adjuvant chemotherapy was administered less frequently in women with breast cancer and diabetes (van de Poll-Franse *et al.* 2007). Alongside differences in the treatment itself, women with diabetes have a lower likelihood of completing chemotherapy (Bekele *et al.* 2024), possibly due to the increased risk of chemotherapy toxicity and complications leading to hospitalisation.

Previous studies (Supplementary Table 1) indicate that all-cause mortality is higher in breast cancer patients with diabetes compared to cancer-specific mortality. This may be because those with diabetes are predisposed to various other conditions that can worsen their prognosis, with Lawrenson *et al.* reporting that 82% of women with diabetes had at least one comorbidity in their study (Lawrenson *et al.* 2024). Consequently, factors other than breast cancer itself may primarily drive the poorer outcomes in breast cancer patients with T2DM. One significant factor could be cardiovascular diseases (CVD) such as ischaemic heart disease and stroke, as T2DM increases the risk of CVD mortality by 18% (Raghavan *et al.* 2019). Furthermore, few prior studies have taken into account potential confounders linking T2DM and cancer, such as age, BMI and smoking, which could contribute to the adverse effects that having T2DM has on breast cancer outcomes.

We have therefore used a real-world data platform to analyse the impact of pre-existing diabetes on outcomes after surgical treatment of localised breast cancer, after correction for comorbidities and confounding factors.

## Methods

We utilised the TriNetX platform (Palchuk *et al.* 2023), a healthcare research organisation based in the United States. We used the platform’s Global Collaborative Network, which contains electronic medical records from approximately 144 million patients across 119 healthcare organisations worldwide. The data available on the TriNetX platform are de-identified per the standard defined in Section §164.514(a) of the Health Insurance Portability and Accountability Act (HIPAA) Privacy Rule. The process by which the data are de-identified is attested to through a formal determination by a qualified expert, as defined in Section §164.514(b)(1) of the HIPAA Privacy Rule. The data we reviewed are a secondary analysis of existing data and do not involve interaction with human subjects. Thus, this retrospective cohort analysis study is exempt from informed consent.

We constructed cohorts of women above the age of 18 with localised breast cancer who underwent surgical treatment between 1 January 2008 and 31 December 2019. There were two groups of cohorts: one consisting of patients diagnosed with T2DM before their breast cancer diagnosis, and the other comprising patients without T2DM before their breast cancer diagnosis. Within each group, there were three cohorts categorised by cancer stage: one including stages 1, 2 or 3; another with either stage 1 or 2; and a third exclusively composed of stage 3 breast cancer patients. We also created a cohort of patients treated with anti-oestrogen treatments as a surrogate for those with ER-positive (ER+) cancer.

- Patients with ICD-10 coded (C50) malignant neoplasms of the breast (excluding those with stage 4 or M1 metastatic cancer) before undergoing breast cancer surgery (either a mastectomy, excision or resection coded under ICD-10, SNOMED and CPT).
  and
- Patients with T2DM (either ICD-10 coded (E11) type 2 diabetes mellitus, HbA1C ≥ 6.5% or in receipt of a glucose-lowering drug coded under RxNorm) and without ICD-10 coded (E10) type 1 diabetes mellitus, before undergoing breast cancer surgery (coded for same as above).

Control cohorts without diabetes were defined in the same way, with the sole difference being the exclusion of patients who had T2DM before receiving breast cancer surgery (using the same compound definition). To examine receipt of adjuvant chemotherapy, we used identical cohorts but extended the time window for inclusion to all those who had surgery before April 2024.

All statistical analysis was conducted in January 2025 on the TriNetX platform, which utilises R, Java and Python programming languages. After selecting cohorts, baseline statistical comparisons between diabetes and non-diabetes cohorts were conducted in pairs based on cancer stage. This included age, ethnicity, smoking status, BMI and vascular disease outcomes.

Propensity score matching for each pair of cohorts under comparison was then performed. This was carried out on the TriNetX platform with an inbuilt 1:1 greedy nearest-neighbour matching algorithm with a caliper of 0.1 pooled standard deviations. This process produced equally sized cohorts between diabetes and non-diabetes groups adjusted for age, ethnicity, nicotine dependence (ICD-10 code F17) as a proxy for smoking status, and BMI. Vascular disease outcomes, including ICD-10 coded acute myocardial infarction (I21), cerebral infarction (I63) and ischaemic heart diseases (I20-25) were included in the propensity matching where indicated.

The day of breast cancer surgery was designated as the index event. The baseline time window of characteristics considered in the propensity score matching includes all the historical data of the patients available in TriNetX before the index event. For OS, patient mortality was based on TriNetX coding for ‘deceased’ or under ICD-10 (R99) for ‘ill-defined and unknown cause of mortality’, starting from the day after surgery until 18 years later. Kaplan–Meier curves, alongside HRs with 95% confidence intervals (CI), were generated. Adjuvant chemotherapy was defined as receipt of any of six cytotoxic treatments, identified by RxNorm coding (epirubicin, cyclophosphamide, paclitaxel, capecitabine, carboplatin, docetaxel). The time window was from the day after surgery until 3 months afterwards.

## Results

From the Global Collaborative Network, the TriNetX database identified 1,488 women who had either stage 1, 2 or 3 breast cancer with T2DM, and 7,284 women who had either stage 1, 2 or 3 breast cancer without T2DM before surgery from January 2008 to December 2019. There were 1,328 women in the cohort with stage 1 or 2 breast cancer with T2DM and 6,707 in the cohort with stage 1 or 2 breast cancer without T2DM. The database returned 202 women for the cohort with stage 3 breast cancer with T2DM and 793 women for the cohort with stage 3 breast cancer without T2DM. The numbers in each cohort before and after matching are summarised in Table 1. Baseline characteristics between the groups are shown in Table 2. Across all cancer stages, people with diabetes were significantly older, more likely to smoke and had a higher BMI.

**Table 1:** Numbers of patients in each cohort before and after matching.

		Diabetes	No diabetes
All stages 1–3
	Unmatched	1,488	7,284
	Matched	1,462	1,462
	Vascular disease matched	1,452	1,452
Stage 1–2		1,328	6,707
	Matched	1,302	1,302
Stage 3		202	793
	Matched	193	191

**Table 2:** Baseline statistics comparison between unadjusted diabetic and non-diabetic cohorts of all breast cancer stages.

Characteristic	T2DM	No T2DM	*P* value
Age at index (years)	63.66 ± 11.14	58.56 ± 12.95	<0.0001
Smoking status	11.56%	6.84%	<0.0001
BMI (kg/m^2^)	33.13 ± 7.80	28.67 ± 6.35	<0.0001
Ethnicity: white	57%	77%	<0.0001
Ethnicity: black	34%	16%	<0.0001
Ethnicity: other	7%	5%	0.0008

**Figure 1:**
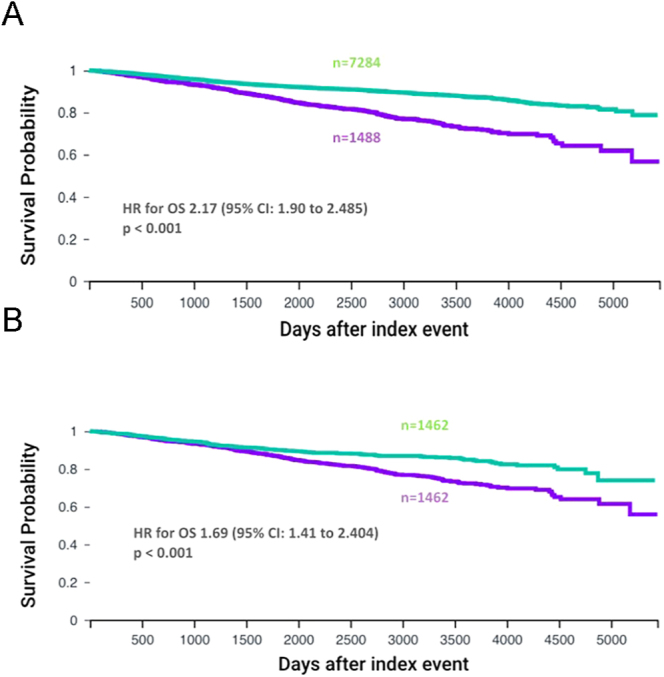
OS for all early cancer stages. Kaplan–Meier curves comparing survival of patients with stage 1, 2 or 3 breast cancer after surgery according to diabetes status. The curves show outcomes before propensity matching (A) and after propensity matching (B) for age, ethnicity, smoking status and BMI. The purple curve represents patients with T2DM and the green curve represents patients without T2DM.

OS was compared between the diabetes and no-diabetes breast cancer cohorts for all early cancer stages (stage 1, 2 or 3) before and after propensity score matching for age, ethnicity, smoking status and BMI. After propensity score matching, there were 1,462 patients in both cohorts. For both comparisons, the index event was breast cancer surgery and the time window was from the day after the surgery onwards indefinitely. Before propensity score matching, the diabetes cohort had statistically worse OS than the no-diabetes cohort (*P* < 0.0001) in both unmatched and matched models. The HR for OS before propensity score matching was 2.17 (95% CI: 1.90–2.485) (Fig. 1A). After propensity score matching for age, BMI and smoking status, this remained significant with a HR of 1.69 (95% CI: 1.41–2.04) (Fig. 1B). Median survival was not reached in either cohort. Median follow-up time was 2,458 days in those with diabetes and 2,460 days in those without diabetes.

**Figure 2:**
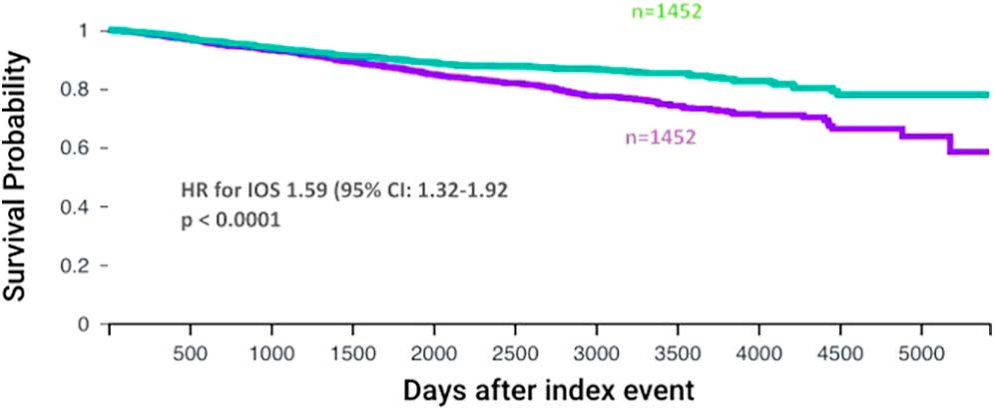
OS for all early cancer stages adjusting for vascular disease outcomes. Kaplan–Meier curves comparing survival of patients with stage 1, 2 or 3 breast cancer after surgery according to diabetes status. The curves show outcomes after propensity matching for age, ethnicity, smoking status, BMI and vascular diseases (cerebral infarction, myocardial infarction or ischaemic heart disease). The purple curve represents patients with T2DM and the green curve represents patients without T2DM.

After inclusion of cardiovascular disease in the propensity score matching, there were 1,452 patients in both cohorts. OS remained worse than the no-diabetes cohort (*P* < 0.0001) in this model. The HR for OS was 1.59 (95% CI: 1.32–1.92) (Fig. 2). Median survival was not reached in either cohort.

**Figure 3:**
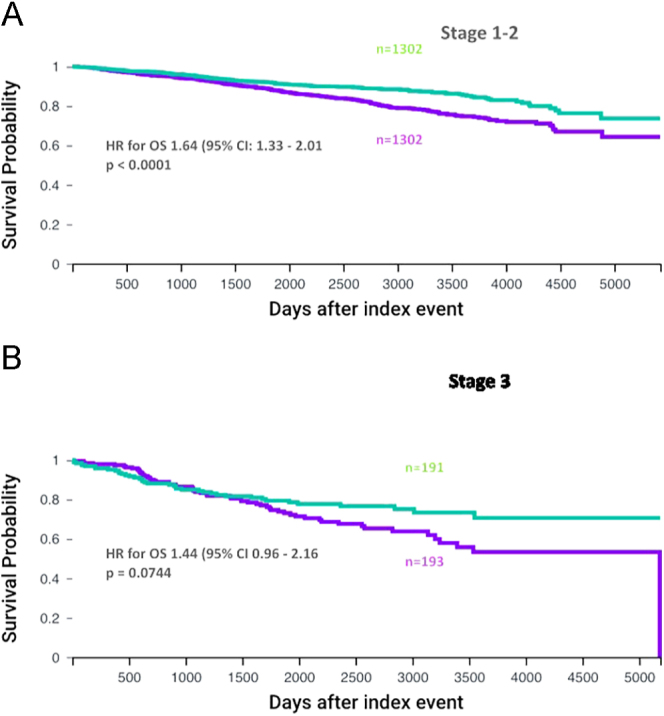
OS stratified by cancer stage. Kaplan–Meier curves comparing survival of patients with stage 1 or 2 breast cancer (A) or stage 3 breast cancer (B) after surgery according to diabetes status. The curves show outcomes after propensity matching for age, ethnicity, smoking status and BMI. The purple curve represents patients with T2DM and the green curve represents patients without T2DM.

We then compared OS in diabetes and no-diabetes cohorts stratified by cancer stage. Among those with stage 1 or 2 breast cancer (*n* = 1,302 for both diabetes and no-diabetes cohorts after matching) and for stage 3 breast cancer (*n* = 193 for both diabetes and no-diabetes cohorts), after propensity score matching for age, ethnicity, smoking status and BMI (Fig. 3). Those with diabetes had statistically worse OS than the no-diabetes cohort in the stage 1 or 2 breast cancer model (*P* < 0.0001), but differences were not significant in those with stage 3. The HR for OS for the stage 1 or 2 model was 1.64 (95% CI: 1.33–2.01) and for the stage 3 model it was 1.44 (95% CI: 0.96–2.16). Median survival was reached in the stage 3 breast cancer diabetes cohort (5,182 days) but not for the no-diabetes cohort or either of the stage 1 & 2 breast cancer cohorts.

**Figure 4:**
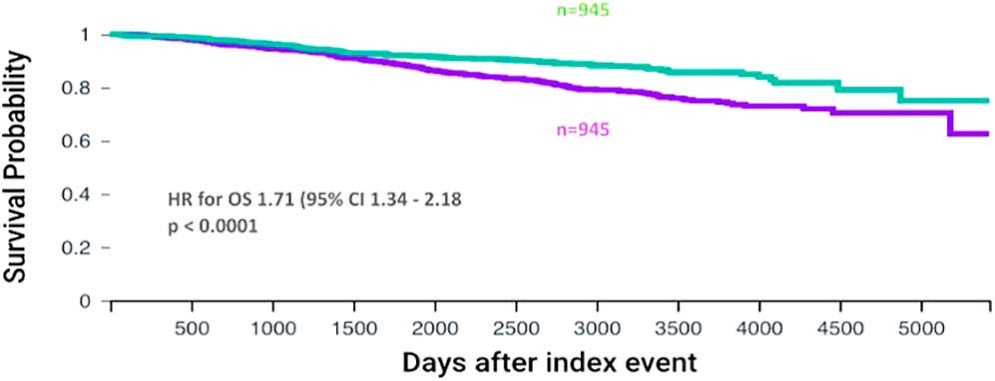
OS for all early cancer stages limited to those treated with an anti-oestrogen or aromatase inhibitor drug. Kaplan–Meier curves comparing survival of patients with stage 1, 2 or 3 breast cancer after surgery according to diabetes status, in those treated with an anti-oestrogen or aromatase inhibitor drug. The curves show outcomes after propensity matching for age, ethnicity, smoking status and BMI. The purple curve represents patients with T2DM and the green curve represents patients without T2DM.

**Figure 5:**
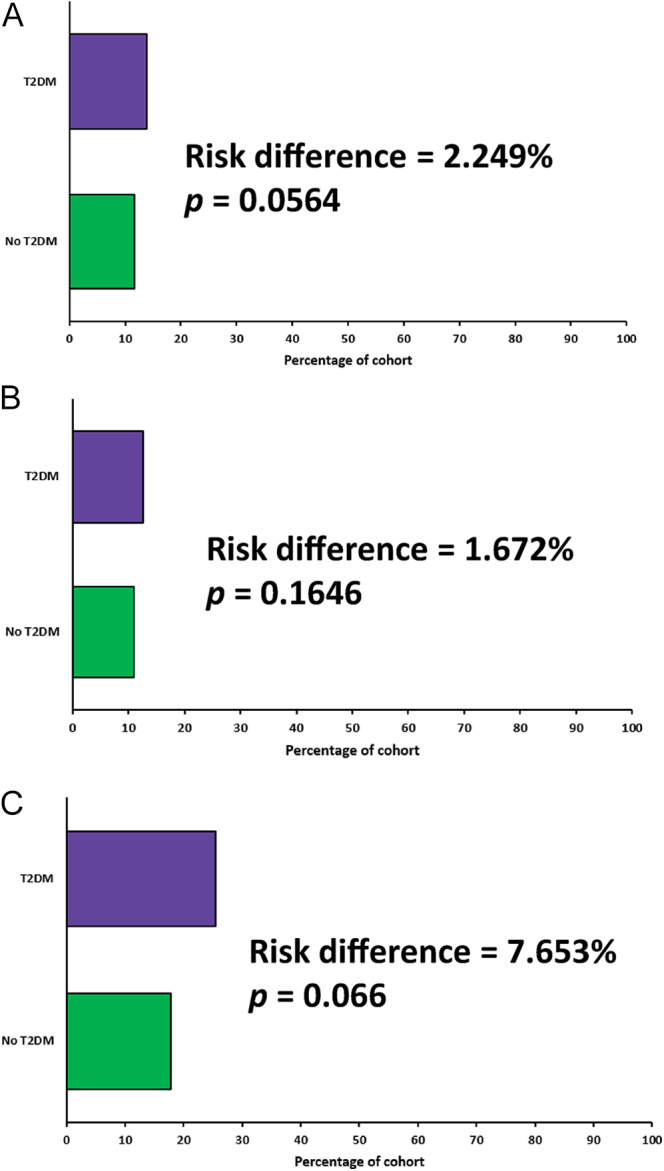
Receipt of adjuvant chemotherapy stratified by cancer stage. Measures of association graphs comparing the rate of receiving adjuvant chemotherapy (defined as chemotherapy started within 3 months of breast cancer surgery) depending on diabetes status for all patients (A), patients with stage 1 or 2 breast cancer (B) and patients with stage 3 breast cancer (C). The cohorts have been propensity matched for age, ethnicity, smoking status and BMI.

**Figure 6:**
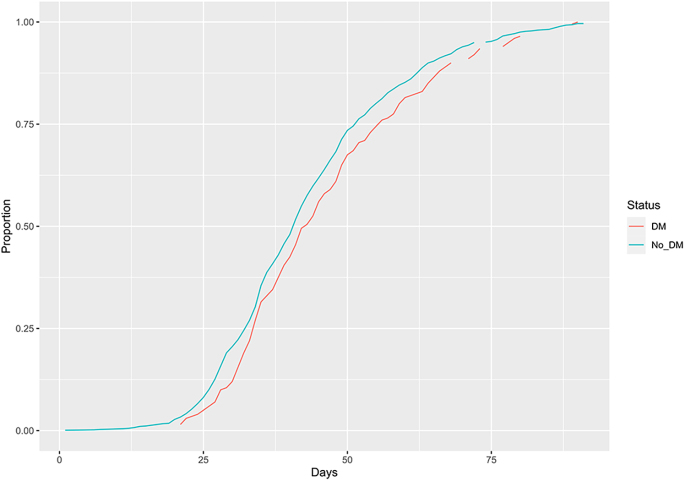
Time to starting adjuvant chemotherapy. Graph showing time from surgery to starting adjuvant chemotherapy, according to presence or absence of diabetes.

We then examined OS in those treated with anti-oestrogen therapy (including aromatase inhibitors), as a surrogate for ER+ disease. After matching, 945 patients were included. OS in those with diabetes remained worse than the no-diabetes cohort (*P* < 0.0001) in this model. The HR for OS was 1.71 (95% CI: 1.34–2.18) (Fig. 4). Finally, we compared the rate of receiving adjuvant chemotherapy between women with and without diabetes stratified by cancer stage, after propensity score matching for age, ethnicity, smoking status and BMI. There were 1,601 patients in both all-stages cohorts, 1,435 patients in both stage 1 or 2 cohorts and 196 patients in both stage 3 cohorts. There was no significant difference in the proportion of patients receiving adjuvant chemotherapy between those with and without diabetes (Fig. 5). Nor was there a significant difference in the number of days from surgery to receipt of first dose of chemotherapy (Fig. 6), with a median of 41 days in those without diabetes and 43 days in those with diabetes (*P* = 0.054).

## Discussion

In this retrospective study, we found that among women with localised breast cancer, those with T2DM experience significantly worse OS compared to their non-diabetes counterparts, reinforcing existing evidence in the literature (Mu *et al.* 2017, Lee *et al.* 2019). Crucially, we were able to correct for differences in age, BMI, smoking status and prevalence of cardiovascular disease, and the persistence of a significant difference in OS after propensity score matching underscores the substantial impact of diabetes itself on patient survival.

In a recent study, Lawrenson *et al.* observed a similar trend of worse survival in diabetes, with survival rates decreasing as cancer stages progressed (Lawrenson *et al.* 2024). Interestingly, Lawrenson *et al.* found that most deaths in their diabetes cohort were due to causes unrelated to breast cancer, with CVD being the leading cause of death (Lawrenson *et al.* 2024). After adjusting for confounders, including age, cancer stage and grade, ethnicity, deprivation and cancer treatment, the HR for breast cancer-specific mortality in those with diabetes was 0.99 (95% CI: 0.89–1.11) (Lawrenson *et al.* 2024). This contrasts with our results, where, although we could not report cause-specific mortality, we used propensity score matching to balance cohorts both for the key risk factors (age, BMI and smoking) but also for the presence of cardiovascular disease, and found a persistent poorer OS.

However, the studies did use different methodology. Lawrenson *et al.* calculated a C3 index score, a cancer-specific measure of patient comorbidity (Sarfati *et al.* 2014), due to a lack of information on BMI and smoking status (Lawrenson *et al.* 2024), whereas this study used propensity scoring to balance cohorts before analysis.

Vascular disease is a critical confounder in studies of survival among those with diabetes, given its significant contribution to mortality in this group (Dal Canto *et al.* 2019). Unfortunately, few studies account for vascular disease outcomes when assessing the impact of diabetes on cancer, which hampers our understanding of the direct effects of diabetes on cancer outcomes. Therefore, our study’s finding of significantly worse OS in women with diabetes, even after propensity score matching for vascular diseases, is particularly noteworthy. This evidence supports the notion that diabetes directly influences cancer outcomes rather than indirectly worsening prognosis through secondary mechanisms such as cardiovascular disease.

This difference in OS after propensity score matching was maintained in those with early and later-stage localised cancers. As expected, the OS in stage 3 breast cancer patients was worse compared with the stage 1 or 2 breast cancer cohort regardless of diabetes status. However, the worse OS in those with stage 3 cancers and diabetes compared to stage 3 cancers without diabetes did not reach statistical significance, likely due to the small number of patients in this cohort.

We did not observe a reduced rate of adjuvant chemotherapy receipt in those with diabetes or any evidence of delays in starting chemotherapy. This differs from some prior reports (van de Poll-Franse *et al.* 2007, Bekele *et al.* 2024). Notably though, Bekele *et al.* focused on treatment of women from low-income groups. In contrast, the TriNetX database predominantly draws on data from academic health centres, and this may account for some differences in practice. Notably, we were only able to examine time to starting treatment. We were not able to compare rates of completion of chemotherapy or delays between doses.

The strengths of this study include the utilisation of the TriNetX platform, with coded data on a high number of patients. This has allowed propensity score matching to account for numerous potential confounders, in contrast to many prior studies. To maximise accuracy, we utilised inclusion criteria incorporating multiple coding systems and used a broad composite definition of diabetes. We also limited the analysis to those with coded stage of cancer and receipt of surgery to ensure accuracy.

However, there are also some important limitations. We did not have access to data regarding cause of death. Therefore, although we included pre-existing cardiovascular disease in the propensity score model, it is not possible to fully attribute remaining increased risk to cancer-specific causes. Propensity score matching itself does have limitations, as it only controls for known confounders that are included in the model, leaving potential biases from unmeasured confounders (Yao *et al.* 2017), and simulates randomisation by controlling for multiple confounding variables simultaneously (Wang 2021). In addition, in cases where suitable matches were not available, data from unmatched patients were discarded, which led to a reduced effective sample size. Any study that relies on routinely collected clinical codes is liable to error due to incorrect coding and missing confounders’ data. Although we restricted our inclusion criteria to specifically coded cancer stages, this may have introduced bias, as some centres contributing data may have more comprehensive staging reporting than others and thus may not be representative of all hospitals. In addition, the hospitals inputting data onto TriNetX are more likely to be academic health centres, which typically have better access to endocrinologists and provide guideline-adherent treatment. A further limitation is that as TriNetX is a dynamic dataset drawing from EHRs over time, participants may appear in more than one group. For example, if a patient was diagnosed with stage 1 cancer but subsequently developed stage 3 disease, they would appear in both cohorts but, to avoid duplication, would only be included in the stages 1–3 cohort as a single patient. Thus, the numbers of patients in the sub-cohorts can be higher than the number in the all-stages cohort, as in this study. However, as comparisons were between those with and without diabetes, not between stages, this is unlikely to have affected the results.

A further limitation is that we limited this analysis to receipt of adjuvant chemotherapy and did not examine receipt of neo-adjuvant treatment in this analysis. We were also unable to evaluate differences in hormone and HER-2 status between the groups, as this was not robustly coded from all centres, which could contribute to differences in outcomes. However, we were able to construct a cohort of patients who had received treatment with either oestrogen receptor blocking drugs or aromatase inhibitors as a surrogate for those with ER+ cancers. This cohort showed a similar impact of diabetes on OS, suggesting the impact of diabetes is not dependent on oestrogen receptor status.

Future research should explore other potential mechanisms behind T2DM’s effect on breast cancer that were not covered in this study. Studies utilising cause-specific mortality are critical, as is matching with accurate cancer data such as TNM status, hormonal status and grade. This could help determine whether the poorer OS in patients with diabetes is due to diabetes’ impact on breast cancer or potential collider bias if diabetes is associated with more aggressive cancers, which would naturally lead to worse OS. In addition, examining treatment continuation could provide insights into delays and/or completion rates of chemotherapy in breast cancer patients with diabetes, as our study only tracked the initiation of adjuvant chemotherapy. Another important area of investigation is the effect of glycaemic control on cancer outcomes. Comparing the outcomes of patients with poor versus good glycaemic control could reveal whether tighter glycaemic control can improve OS in breast cancer patients with diabetes.

In summary, our results indicate that T2DM is associated with poorer OS across all stages of localised breast cancer post-surgery, even after adjusting for age, ethnicity, BMI and smoking status. Importantly, this trend persisted even after adjusting for vascular disease outcomes. We found no difference in rates of starting adjuvant chemotherapy to account for this. This suggests that T2DM may have a direct impact on the outcomes of patients with breast cancer. Further work is necessary to fully understand the mechanism of this effect in order to guide interventions to improve outcomes.

## Supplementary materials



## Declaration of interest

AK, NH and DM have no conflicts of interest to declare. GH and GS are employed by TriNetX.

## Funding

This work did not receive any specific grant from any funding agency in the public, commercial or not-for-profit sector.

## Author contribution statement

DM and NH conceived the study. AK constructed the cohorts and performed analysis under guidance from GH, GS and DM. All authors wrote the paper.
