# Supplementary material for: Using routinely collected patient data to study the impact of type 2 diabetes on breast cancer
Source: Endocr Oncol. 2025 Jul 8;5(1):e240039. doi: 10.1530/EO-24-0039 (PMC12243097; doi:10.1530/EO-24-0039)
Supplement: Supplementary file 1 [file supplementary_materials.pdf]

Supplementary Table 1: Outcomes of breast cancer patients with T2DM compared with non-diabetic controls across recent studies

Citation	Outcome(s) measured
(Mu et al., 2017)	Significantly shorter 5-year disease-free survival (DFS) than non-diabetic patients (78.8% vs 87.7%), significantly shorter 5-year overall survival (OS) than non-diabetic patients (85.5% vs 92.5%)
(Sonnenblick et al., 2017)	Hormone receptor-positive patients not treated with metformin experienced worse DFS (multivariable hazard ratio [HR] = 1.40), distant disease-free survival (DDFS) (multivariable HR = 1.56) and OS (multivariable HR of 1.87)
(Lega et al., 2018)	All-cause mortality adjusted HR of 1.11
(Chang et al., 2018)	Poorly controlled glycaemia in diabetic patients (defined as mean HbA1C >9%) had significantly higher risks for all cause mortality (HR = 3.65) and breast cancer-specific mortality (HR = 8.37) compared to non-diabetic women
(Shao et al., 2018)	Mortality adjusted HR of 1.17
(Lee et al., 2019)	Following targeted therapy: disease free survival HR of 4.5, significantly shorter disease-free survival (DFS) compared with non-diabetic patients (74.1% vs 91.9%), significantly shorter overall survival (OS) compared with non-diabetic patients (91.7% vs 99.1%)
(Tao et al., 2020)	All-cause mortality HR of 1.31
(Escala-Garcia et al., 2020)	Cancer-specific mortality hazard ratio (HR) of 1.10
(Sheppard et al., 2020)	Mortality adjusted HR of 1.87
(Tobe et al., 2022)	Significantly shorter distant metastasis-free survival
(Lawrenson et al., 2024)	Cancer-specific 5 year survival rate of 87% for diabetics vs 89% for non-diabetics and 10 year survival rate of 79% for diabetics vs 84% for non diabetics
